# Inhibition of CNOT2 Induces Apoptosis via MID1IP1 in Colorectal Cancer Cells by Activating p53

**DOI:** 10.3390/biom11101492

**Published:** 2021-10-10

**Authors:** Ji Hoon Jung, Duckgue Lee, Hyun Min Ko, Hyeung-Jin Jang

**Affiliations:** 1 College of Korean Medicine, Kyung Hee University, Seoul 02447, Korea; kocic77@khu.ac.kr (H.M.K.); hjjang@khu.ac.kr (H.-J.J.); 2 Soonchunhyang Institute of Medi-bio Science (SIMS), Soonchunhyang University, Cheonan-si 31151, Korea; tomyoo27@gmail.com

**Keywords:** CNOT2, p53, apoptosis, doxorubicin, colorectal cancer cell

## Abstract

CCR4-NOT transcription complex subunit 2 (CNOT2), a subunit of the CCR4-NOT complex, has been described in cancer progression. The CNOT complex plays an important role in multiple cellular functions. Recent studies in our laboratory showed that CNOT2 promotes breast cancer cell proliferation and angiogenesis. In addition, CNOT2 signals are critically related to apoptosis induced by atorvastatin in lung cancer cells. Furthermore, depletion of CNOT2 was shown to enhance the antitumor effect of midline 1 interacting protein 1 (MID1IP1) depletion, thus inhibiting c-Myc expression in liver cancer cells. However, the molecular mechanisms related to its oncogenic role remain unclear. Herein, for the first time, we report that CNOT2 inhibition can induce apoptosis in colorectal cancer cells by activating p53. Inhibition of CNOT2 markedly induced apoptosis in various cancer cells like that of the wild-type p53. Furthermore, inhibition of CNOT2 elongated p53 s half-life. Previously, our laboratory demonstrated that MID1IP1 promoted colocalization with c-Myc mediated by CNOT2. Interestingly, inhibition of CNOT2 cannot induce p53 expression without MID1IP1 or apoptosis in cancer cells. In conclusion, our results demonstrate that CNOT2 inhibition induces apoptosis through MID1IP1 by activating p53.

## 1. Introduction

The CCR4-NOT complex (CNOT) consists of 11 subunits and is a regulator of transcription and translation [1,2]. The CNOT complex plays a particularly important role in multiple functions in terms of regulating translation, mRNA stability, and RNA polymerase I and II transcriptions [3]. Moreover, the CCR4-NOT complex contributes to the repression of MHC class II transcription [4]. CNOT consists of nine subunits: CNOT1, CNOT2, CNOT3, CNOT6, CNOT6L, CNOT7, CNOT8, CNOT9, and CNOT10 [5]. Recent studies showed that inhibition of CNOT2 in human cancer cells inhibits cancer cell proliferation and angiogenesis through VEGF signaling in cancer cells, suggesting that CNOT2 acts as an oncogene [6,7,8]. Nevertheless, the molecular mechanisms related to CNOT2 still remain unclear.

p53, a tumor suppressor, is activated by various stressors, and the p53 transcriptional regulator is able to induce or repress numerous target genes’ transcription [9]. p53 can prevent tumorigenesis by regulating metabolism, inhibiting metastasis and cell proliferation, and inducing apoptosis. The p53 tumor suppressor can prevent tumor formation by inducing cellular senescence, stopping cell proliferation, regulating EMT, and inducing apoptosis in various human cancers [9,10]. For example, the p53 target genes p21, DDB2, GADD45A, and BTG2 are involved in cell cycle arrest and DNA repair. Furthermore, p53 can induce antioxidant genes, for example, GLS2 and TIGAR. These genes eliminate reactive oxygen species (ROS) [11]. In addition, p53 can also upregulate the expression of multiple apoptotic genes, such as PUMA, Bax, and Noxa, which play key roles in p53-mediated apoptosis [12,13]. A number of target genes involved in p53-dependent apoptosis have been studied in depth; however, the p53-regulated pathway is not fully understood.

Our latest study showed that CNOT2 is involved in apoptotic cell death caused by atorvastatin in non-small cell lung cancers [7]. Furthermore, CNOT2 is related to c-Myc, which is mediated by the ribosomal proteins L5, L11, or by midline 1 interacting protein 1 (MID1IP1) in liver cancer cells [6]. In this paper, depletion of MID1IP1 inhibits c-Myc expression; however, loss of L5 or L11 rescued c-Myc in MID1IP1 deleted in Hep2 and Huh7 cells. Furthermore, CNOT2 depletion enhanced the anticancer effect to reduce c-Myc expression. It is well known that the ribosomal proteins L5 and L11 interact with MDM2 and regulate p53 expression [12,13,14]. These studies led us to believe that CNOT2 probably affects other oncogenes or tumor suppressor genes to control cancer cells, such as c-Myc or p53.

In this study, we identified CNOT2 as a new regulator of apoptosis and proliferation by activating p53 via MID1IP1 in cancer cells. Knockdown of CNOT2 caused apoptosis in p53 wild-type cancer cells, but not in p53 null-type cancer cells. Interestingly, CNOT2 is amplified in various human cancers, and the knockdown of CNOT2 is related to p53 in cancer cells. Thus, we wrote in the initial report that CNOT2 can induce p53-dependent apoptosis and inhibit cancer cell proliferation by activating p53 via MID1IP1.

## 2. Materials and Methods

### 2.1. Cell Culture

HCT116^p53+/+^ (human colorectal cancer cell line, wild-type p53), HCT116^p53−/−^ (p53 knockout), SW480 (human colorectal cancer cell line), SW620 (human colorectal cancer cell line), and HCT15 (human colorectal cancer cell line) cell lines were grown in RPMI1640 medium (WelGENE, Gyeongsan, Korea) supplemented with 10% fetal bovine serum (FBS) (WelGENE, Gyeongsan, Korea) and 1% antibiotic solution (100 units/mL penicillin and 100 μg/mL streptomycin) in 5% CO_2_ at 37 °C. The cell culture was performed as described in a previous paper [15].

### 2.2. RNA Interference and Plasmid

On the basis of Jung’s paper [15], HCT116^p53+/+^ cells were seeded in a 6-well plate at 7 × 10^4^ overnight. Thereafter, they were transfected with CNOT2 siRNA (Cat.No.4848-1) (Bioneer, Daejeon, Korea), p53 siRNA (Cat.No.7157-1) (Bioneer, Daejeon, Korea), MID1IP1 siRNA (Cat.No.58526-1) (Bioneer, Daejeon, Korea) or control siRNA (Cat.No.SN-1003) (Bioneer, Daejeon, Korea). HA-CNOT2 was kindly provided by Dr. KS Kim (Yonsei University, Seoul, Korea). The transfection assay was performed as described in Jung’s paper [15].

### 2.3. Cell Viability

The cell viability assay was performed as described in Jung’s paper [13]. The cell viability assay was performed using a CCK8 assay kit (Dojindo, Rockville, MD, USA). Cells were counted and seeded into a 96-well plate at a density of 7 × 10^3^ cells/well and incubated overnight in 5% CO_2_ at 37 °C. The absorbance of the samples was measured at 450 nm using a microplate reader. The viability was calculated with optical density (OD) values as a percentage of viable cells in the CNOT2 siRNA transfected group versus control siRNA. All assays were conducted independently three times.

### 2.4. Western Blot Analysis

The Western blotting was performed according to Jung et al.’s paper [13]. Cells were washed in 1XPBS, lysed in NP-40 buffer, and centrifuged at 13,000 rpm for 20 min at 4 °C. The proteins were measured using the Bio-Rad Protein Assay. Thereafter, 20 μg of proteins were separated using SDS-PAGE (8–12%). Furthermore, they were transferred onto Hybond-enhanced chemiluminescence (ECL) transfer membrane (Amersham Pharmacia, Piscataway, NJ, USA). The membranes were blocked in 1 × PBS + 0.1% Tween 20 solutioncontaining 3% skim milk. The membranes were incubated with various antibodies against CNOT2 (1:1000) (#34214), PARP (1:1000) (#9532), p21 (#2947, Cell signaling Technology Inc., Danvers, MA, USA), p53 (DO-1) (1:1000) (SC-126), HA- (SC-7392, Santa Cruz Biotechnologies, Santa Cruz, CA, USA), MID1IP1 (15764-1-AP, ProteinTech Antibody Group, Chicago, IL, USA), and β-actin (1:3000) (A5316, Sigma Aldrich Co., St. Louis, MO, USA). All primary antibodies were diluted in 1XPBS + 0.1% Tween 20 solution. Secondary antibodies were diluted in 1XPBS + 0.1% Tween 20 solution containing 3% skim milk. Protein expression was detected using an enhanced chemiluminescence system (Amersham Pharmacia, Piscataway, NJ, USA) by film.

### 2.5. p53 Stability Assay Using Cycloheximide

On the basis of Jung et al.’s paper [6], HCT116^p53+/+^ cells were transfected with CNOT2 siRNA or negative control siRNA for 72 h. Thereafter, cells were treated with 50 μg/mL cycloheximide for different time points (0, 15, 30, 60 min) and were confirmed using Western blotting with antibodies of p53, p21, CNOT2, and β–actin.

### 2.6. Statistical Analysis

Data were presented as means ± standard deviation (SD). A Student’s t-test for two-group comparison and one-way analysis of variance (ANOVA) followed by a Tukey’s post-hoc test were conducted for multi-group comparison using the GraphPad Prism software (Version 5.0, San Diego, CA, USA). Significant differences were considered if the *p*-value was less than 0.05. All experiments were conducted three times.

## 3. Results

### 3.1. CNOT2 Is Overexpressed in Colon Cancer Patients and Cancer Cells

**Figure 1:**
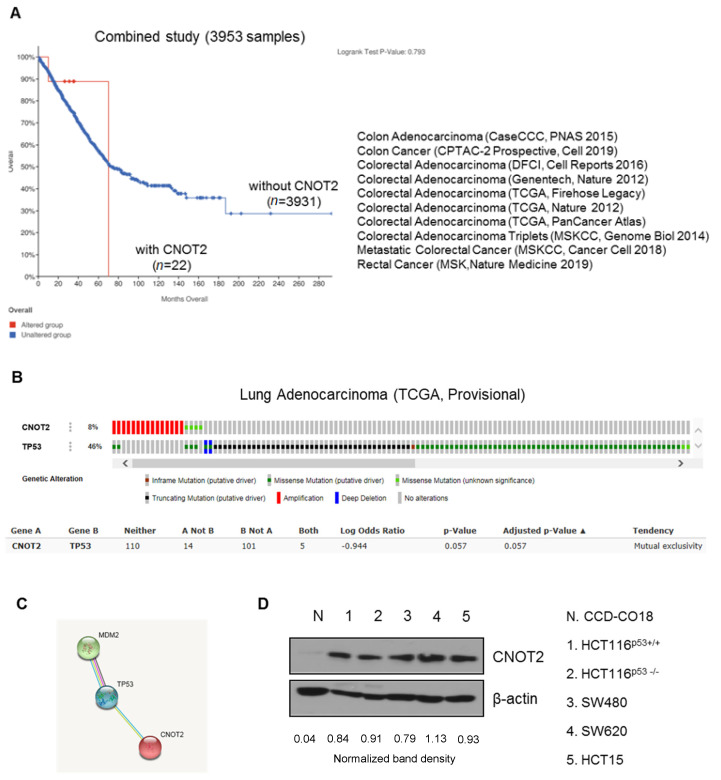
CNOT2 expression in cancer cells. (**A**) TCGA databases, modified from the cBioPortal for Cancer Genomics (http://www.cbioportal.org, accessed on 10 August 2020). (**B**) Analysis of mutual exclusivity was retrieved from cBioPortal and gene alteration status of CNOT2 and TP53 of individual samples. (**C**) Protein–protein interactions, wherein STRING v10 predicts binding between MDM2, p53, and CNOT2. (**D**) CNOT2 protein level in various colon cancer cells was assayed using Western blotting.

According to a recent paper, CNOT2 is associated with lung cancer cells [6,7]. However, there is no paper on the specific role of CNOT2 in colon cancer yet. First of all, we checked the expression of CNOT2 in cancer patient data from cBioportal. The TCGA analysis gave differing results for the sample group, with CNOT2 being overexpressed and nonexpressed. The survival rate was significantly increased in colon cancer patients with low CNOT2 expression as compared with patients with high expression (Figure 1A). Next, we checked whether CNOT2 was related to oncogenes or tumor suppressors. Upregulation of CNOT2 exhibited an inverse relationship to TP53 in lung adenocarcinoma, according to the cBioPortal database analysis (Figure 1B). These results demonstrate that CNOT2 may play an oncogenic role through the inactivation of p53 in different types of cells. Interestingly, upregulation of CNOT2 was significantly correlated with mouse double minute 2 homolog (MDM2) (Figure 1C). Consistently, CNOT2 was overexpressed in cancer cells as compared to normal cells (control) according to Western blotting (Figure 1D). In summary, the results demonstrate that CNOT2 might play an oncogenic role in human cancer cells through the inactivation of p53.

### 3.2. Knockdown of CNOT2 Induces Apoptosis in Cancer Cells by Activating p53

**Figure 2:**
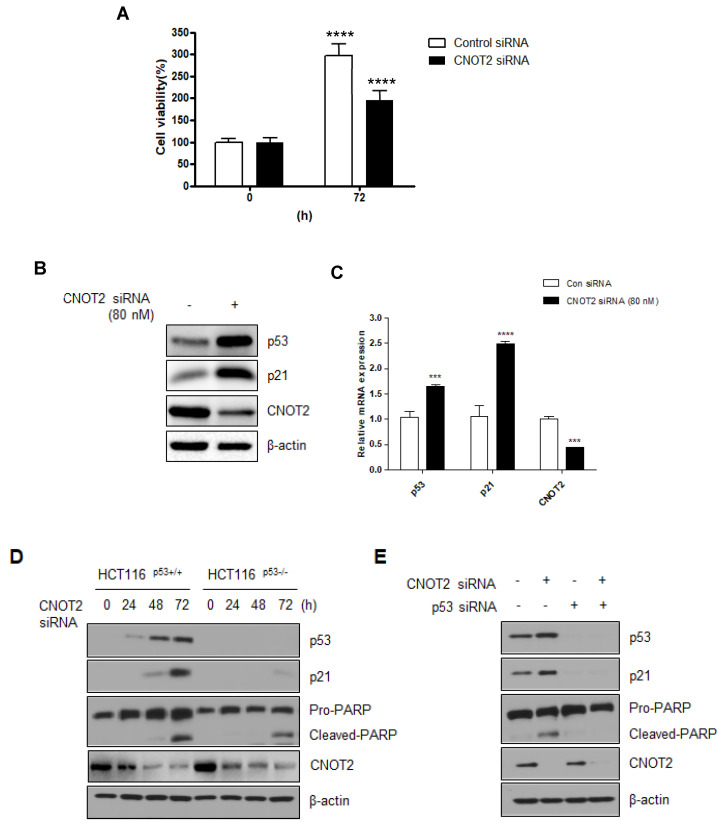
CNOT2 knockdown induces apoptosis by activating p53. (**A**) HCT116^p53+/+^ cells were transfected with CNOT2 or scrambled siRNA for 72 h. Cell viability was evaluated by CCK8 assay (Three independent experiments). (**B**,**C**) HCT116^p53+/+^ cells were transfected with CNOT2 or scrambled siRNA for 72 h. After then, cells were harvested for western blotting or qRT-PCR. (**D**) HCT116^p53+/+^ and HCT116^p53−/−^ cells were transfected with CNOT2 or scramble siRNA at various time points transfection for western blotting with p53, p21, PARP, and β-actin antibodies. (**E**) HCT116^p53+/+^ cells were transfected with (Marked as +) CNOT2 siRNA with or without (Marked as -) p53 siRNA after then harvested 72 h post-transfection for western blotting with indicated antibodies. * p value indicates a significant difference between the groups *** *p* < 0.005, **** *p* < 0.001.

The above results suggest that CNOT2 was probably associated with p53. Moreover, our studies show that the inhibition of CNOT2 expression regulates EMT in breast cancer cells [8]. In addition, CNOT2 involved with atorvastatin induced both apoptotic and autophagic cell death in lung cancer cells [7]. This paper showed that atorvastatin treatment attenuated p53 and decreased CNOT2 expression in a dose-dependent manner. The data in this paper suggest that CNOT2 may be involved in regulating p53 expression in cancer cells. In an attempt to address this, we tested p53 expression through the knockdown of CNOT2 in HCT116^p53+/+^ and HCT116^p53−/−^ cells. First of all, we checked the CNOT2 is related to cancer cell viability using CNOT2 siRNA. As shown in Figure 2A, knockdown of CNOT2 inhibits cancer cell viability compared with the control group. We measured cell viability after inhibiting the expression of CNOT2 using CNOT2 siRNA to confirm that CNOT2 is related to cancer cell viability. As shown in Figure 2B, knockdown of CNOT2 induced not only p53 but also p21 expression. Furthermore, CNOT2 knockdown induced p53 and p21 mRNA levels performed by qRT-PCR (Figure 2C). By performing a Western blotting assay, we found that CNOT2 knockdown induced p53 expression and the expression of its target gene, p21, in a time-dependent manner in HCT116 ^p53+/+^ cells. Interestingly, knockdown of CNOT2 induced the apoptosis marker cleaved PARP in cancer cells containing wild-type p53, and partly induced apoptosis in p53-null cancer cells in a time-dependent manner (Figure 2D). Knockdown of CNOT2 induced p53, p21, and cleaved PARP; however, p53 knockdown by siRNA with CNOT2 siRNA reversed this result in HCT116^p53+/+^ cells (Figure 2E). 

### 3.3. Knockdown of CNOT2 Induces p53 Protein Stability

**Figure 3:**
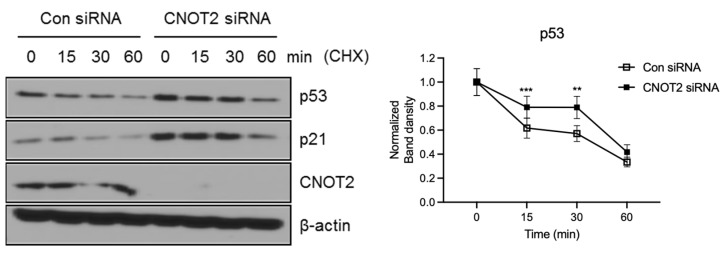
CNOT2 knockdown inhibits p53 degradation. p53 s half-life was increased by CNOT2 knockdown in HCT116^p53+/+^ cells. HCT116^p53+/+^ cells were transfected with CNOT2 or scrambled siRNA and collected 72 h after transfection for Western blotting with indicated antibodies. * p value indicates a significant difference between the groups ** *p* < 0.01, *** *p* < 0.005.

Thereafter, we wanted to know how CNOT2 regulates the p53 protein level. To explore this, we tested the half-life of p53 in the absence and presence of CNOT2. It was shown that the knockdown of CNOT2 extended the half-life of p53 to a greater extent than scramble siRNA. Consistently, p21, a target gene of p53, also extended the half-life to a greater extent in the CNOT2 knockdown group (Figure 3). This suggests that the knockdown of CNOT2 increases p53 stability. 

### 3.4. Knockdown of CNOT2 Enhances Anticancer Effect with MID1IP1

**Figure 4:**
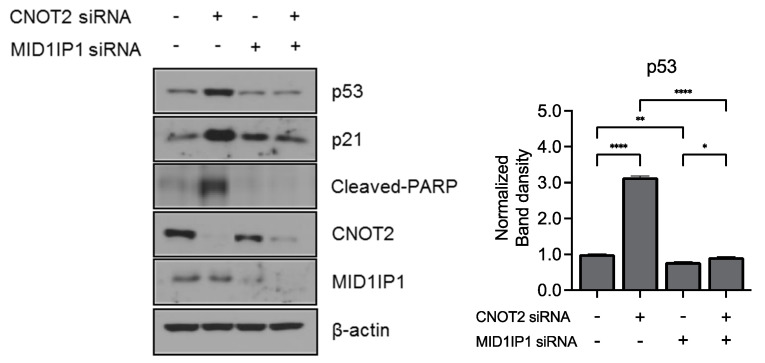
CNOT2 knockdown induces p53 expression via MID1IP1. HCT116^p53+/+^ cells were transfected with CNOT2 siRNA with or without MID1IP1 siRNA, and harvested for Western blotting with the indicated antibodies. * *p* value * *p* < 0.05, ** *p* < 0.01, **** *p* < 0.001.

Our previous studies show that MID1IP1 regulates liver cancer growth through c-Myc mediated by ribosomal protein L5 (RPL5) and L11 (RPL11). Furthermore, CNOT2 knockdown enhances the antitumor effect of MIDIP1 depletion in cancer cells, such as the inhibition of c-Myc, pro-PARP, and pro-caspase3 expression [6]. This study led us to believe that MID1IP1 may be related to p53. To determine whether CNOT2 plays an important role in p53 regulation via MID1IP1, we performed CNOT2 and MID1IP1 knockdown in HCT116^p53+/+^ cells. As shown in Figure 4, knockdown of CNOT2-induced p53, p21, and the cleaved PARP, and knockdown of MID1IP1 by siRNA reversed this induction in HCT116^p53+/+^ cells. These results suggest that CNOT2 knockdown induces apoptosis by inducing p53 via MID1IP1.

### 3.5. Knockdown of CNOT2 Potentiated p53 Expression by Doxorubicin in HCT116^p53+/+^ Cells

**Figure 5:**
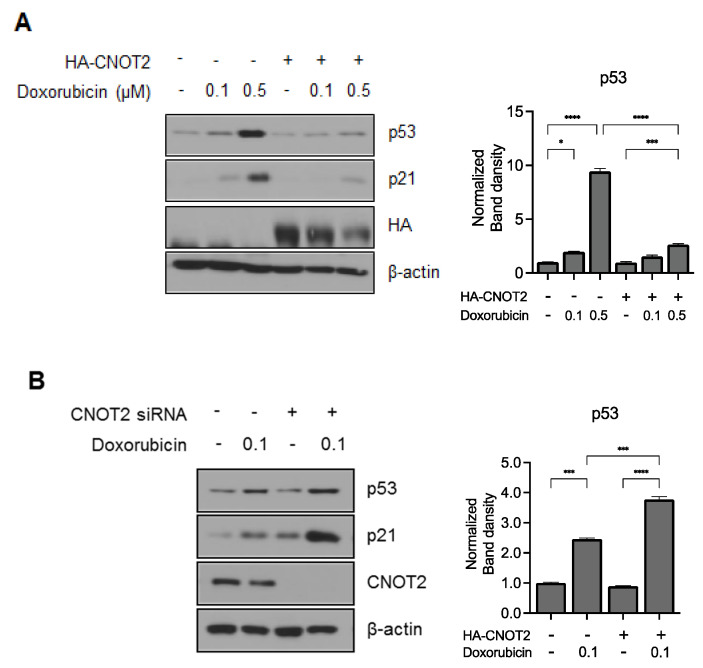
The increased expression of CNOT2 reduces p53 expression, and inhibition of CNOT2 further increases p53 expression due to DNA-damage drugs. (**A**) HCT116^p53+/+^ cells were transfected with HA-CNOT2 or pcDNA for 48 h. After transfection, cells were treated with doxorubicin for 24 h. (**B**) HCT116^p53+/+^ cells were transfected with scramble or CNOT2 siRNA for 48 h. After transfection, cells were treated with doxorubicin for 24 h. * *p* value * *p* < 0.05, *** *p* < 0.005, **** *p* < 0.001.

Doxorubicin, a potent anticancer drug, is most commonly used for various cancer cell lines including colon cancer cells [16]. We determined whether CNOT2 affects the chemical sensitivity of colon cancer cells. Colon cancer is one of the leading causes of death in cancer patients [17]. First, we tested the effect of CNOT2 on p53 expression induced by doxorubicin. Interestingly, overexpression of CNOT2 reduced p53 expression and the expression of its target gene, p21, despite doxorubicin treatment (Figure 5A). Consistently, CNOT2 depletion sensitized p53 expression in HCT116 ^p53+/+^ cells due to doxorubicin, as shown in Figure 5B. These results demonstrate that CNOT2 contributes to cancer cell resistance to anticancer drugs in p53-containing cancer cells, which could be a potential molecular target for chemotherapy in the future.

## 4. Discussion

It is well known that p53 acts as a tumor suppressor gene in cancer cells. Many genes have been identified as being associated with cancer cell growth and the regulation of p53 reactions to apoptosis [15,18,19]. Here, our study showed that CNOT2 is an additional p53 regulation gene in cancer cells. To the best of our knowledge, this study is the first to reveal the functional relationship between CNOT2 and p53 in colon cancer cells.

CNOT2, a subunit of the CCR4-NOT complex, is known to be associated with apoptosis, angiogenesis, autophagy, and metastasis in several types of cancer cells [6,7,8]. We found that the oncogenic role of CNOT2 is supported by the available bioinformatics databases because the high representation of CNOT2 was correlated with a low survival rate in cancer patients. This result demonstrates that CNOT2 acts as a tumor trigger in colon cancer. Intriguingly, both CNOT2 and p53 genes have mutually exclusive properties.

On the basis of this information, we found that CNOT2 knockdown by siRNA is related to decreased colon cancer cell viability. Indeed, our results demonstrate that CNOT2 depletion induces apoptosis in a time-dependent manner. Interestingly, knockdown of CNOT2 induces p53 and its target gene p21. Furthermore, we found that the knockdown of CNOT2 induces p53 in cancer cells. Interestingly, CNOT2 knockdown induces p53-dependent apoptosis in cancer cells. Furthermore, the knockdown of CNOT2 leads to the induction of p53 protein levels.

**Figure 6:**
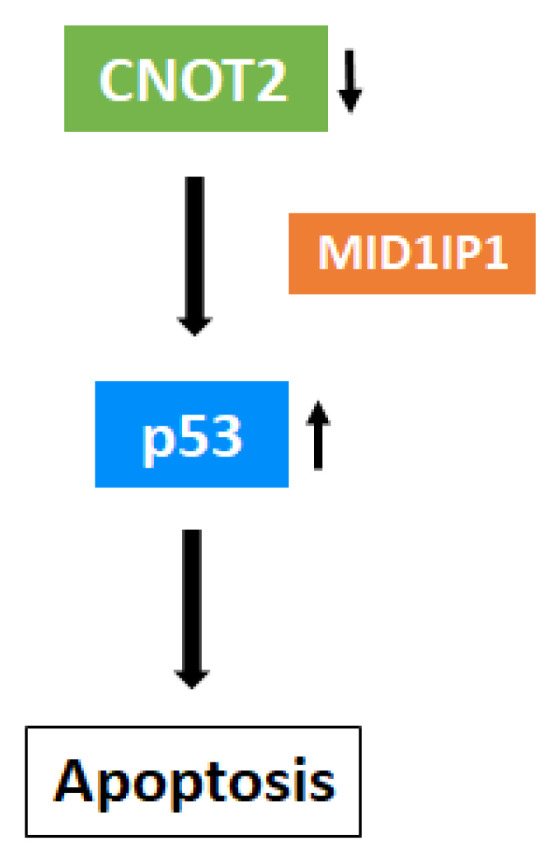
The role of CNOT2 in cancer cells. A mechanistic scheme showing how the inhibition of CNOT2 induces p53 expression and apoptosis. Inhibition of CNOT2 expression induces apoptosis by p53 activation via MID1IP1.

Previous studies showed that MID1IP1 knockdown suppressed c-Myc expression via the ribosomal proteins L5 and L11. Furthermore, MID1IP1 depletion induced p21 expression and increased p21 protein stability [6]. Importantly, MID1IP1 knockdown also inhibited CNOT2 expression. These results suggest that MID1IP1 might be involved in CNOT2 in the regulation of cancer cell growth. As demonstrated herein, our study found a new mechanism related to the role of CNOT2 in cancer, i.e., the activation of p53 via MID1IP1. However, more studies are required to elucidate which pathway is involved in p53 activation, for example, the ribosomal proteins L5 or L11. In summary, the current study demonstrates a new mechanism related to CNOT2 in cancer cells. CNOT2 depletion induces p53 expression via MID1IP1. These findings could be useful for anticancer drug discovery in the future. However, the exact mechanisms involved in the interaction between CNOT2 and p53 in cancer cells remain to be fully explained. Thus, further investigation to this end may show CNOT2 depletion induces p53 (Figure 6).

## Data Availability

Not applicable.
